# Long-term clinical remission in rapidly recurrent maxillary osteosarcoma treated with anlotinib-based multimodal therapy: a case report

**DOI:** 10.1186/s12903-026-08027-w

**Published:** 2026-03-06

**Authors:** Xinru Wang, Jing Wang, Shuping Li

**Affiliations:** 1 The First Clinical Medical College, Gansu University of Chinese Medicine, Lanzhou, 730000 China; 2 Department of Radiation Oncology II, Gansu Provincial Hospital, Lanzhou, 730000 China

**Keywords:** Maxillary osteosarcoma, Local Recurrence, Multimodal Salvage Therapy, Anlotinib, Case Report

## Abstract

**Background:**

Osteosarcoma of the jaw is a rare clinical entity characterized by a high propensity for local recurrence, for which surgical resection serves as the primary treatment modality. However, achieving negative margins in the maxilla is often complicated by the proximity of critical neurovascular and orbital structures. Furthermore, in certain emergency scenarios, surgical compromises may precipitate suboptimal initial control and rapid disease progression. This presents a significant therapeutic dilemma, particularly given the lack of standardized management guidelines for refractory cases.

**Case presentation:**

A 37-year-old male underwent emergency orbital decompression for a vision-threatening maxillary mass. One month later, rapid recurrence near the skull base compressed the optic nerve. Due to the risks of hemorrhage and iatrogenic neural injury from re-biopsy, and the patient’s refusal to accept the risk of jeopardizing his preserved vision, a diagnosis of clinicoradiological recurrence was established based on multiparametric MRI (specifically quantitative ADC analysis) and multidisciplinary consensus. The patient received salvage therapy comprising radiotherapy, chemotherapy, and Anlotinib. Over a 48-month follow-up period, the patient achieved complete radiological remission.

**Conclusions:**

While the emergency intervention successfully preserved vision, it also highlights the risks of deviating from standard oncologic principles, which likely precipitated the rapid recurrence. However, the primary significance of this report is to demonstrate that in refractory cases where salvage surgery is contraindicated, an aggressive multimodal strategy—integrating radiotherapy, chemotherapy, and Anlotinib—may serve as a viable salvage option. These findings warrant further validation in prospective trials.

## Background

Osteosarcoma (OS) is an aggressive malignant neoplasm of mesenchymal origin that predominantly affects the long bones of adolescents and young adults (ages 10–25). It is characterized by its locally invasive nature and high propensity for early systemic metastasis [1, 2]. Osteosarcoma of the jaw (JOS) is a distinct and rare clinical entity, accounting for approximately 6–7% of all osteosarcomas. Unlike long-bone variants, JOS more commonly affects adults aged 30 to 40 years and exhibits a lower propensity for distant metastasis. However, it is characterized by a significantly higher risk of local recurrence. The cornerstone of management is surgical resection with clear margins; however, achieving this in the maxilla is often precluded by the complex craniofacial anatomy and the proximity of critical neurovascular and orbital structures. Consequently, when margins are compromised, the risk of local recurrence increases dramatically, presenting a formidable therapeutic challenge [3–5].

There is no standardized consensus on the management of recurrent or unresectable JOS. Treatment options are typically limited to chemotherapy, radiotherapy, and/or salvage surgery, and reported outcomes remain generally contradictory [6–8].Furthermore, there is a paucity of literature describing long-term success in cases complicated by early, rapid recurrence.

In recent years, targeted therapy has achieved breakthrough effects. Anlotinib has been established as a Class I recommendation by the Chinese Society of Clinical Oncology (CSCO) as a second-line targeted therapy for advanced or unresectable bone and soft tissue sarcomas, with both basic research and clinical reports demonstrating its significant potential in treating advanced osteosarcoma [9–14]. It is important to note that while Anlotinib is approved and widely used in China, it remains an investigational agent for this indication in many other regions. Furthermore, unlike targeted therapies requiring specific mutation screening (e.g., EGFR inhibitors), Anlotinib targets broad angiogenic pathways (VEGFR, FGFR, PDGFR) that are universally upregulated in osteosarcoma [15]. This mechanism allows for its initiation without mandatory pre-treatment molecular profiling. This report presents a case of maxillary osteosarcoma with early, rapid local recurrence that achieved a clinical remission of approximately 48 months through a multimodal regimen integrating Anlotinib, chemotherapy, and radiotherapy.

## Case presentation

**Fig. 1:**
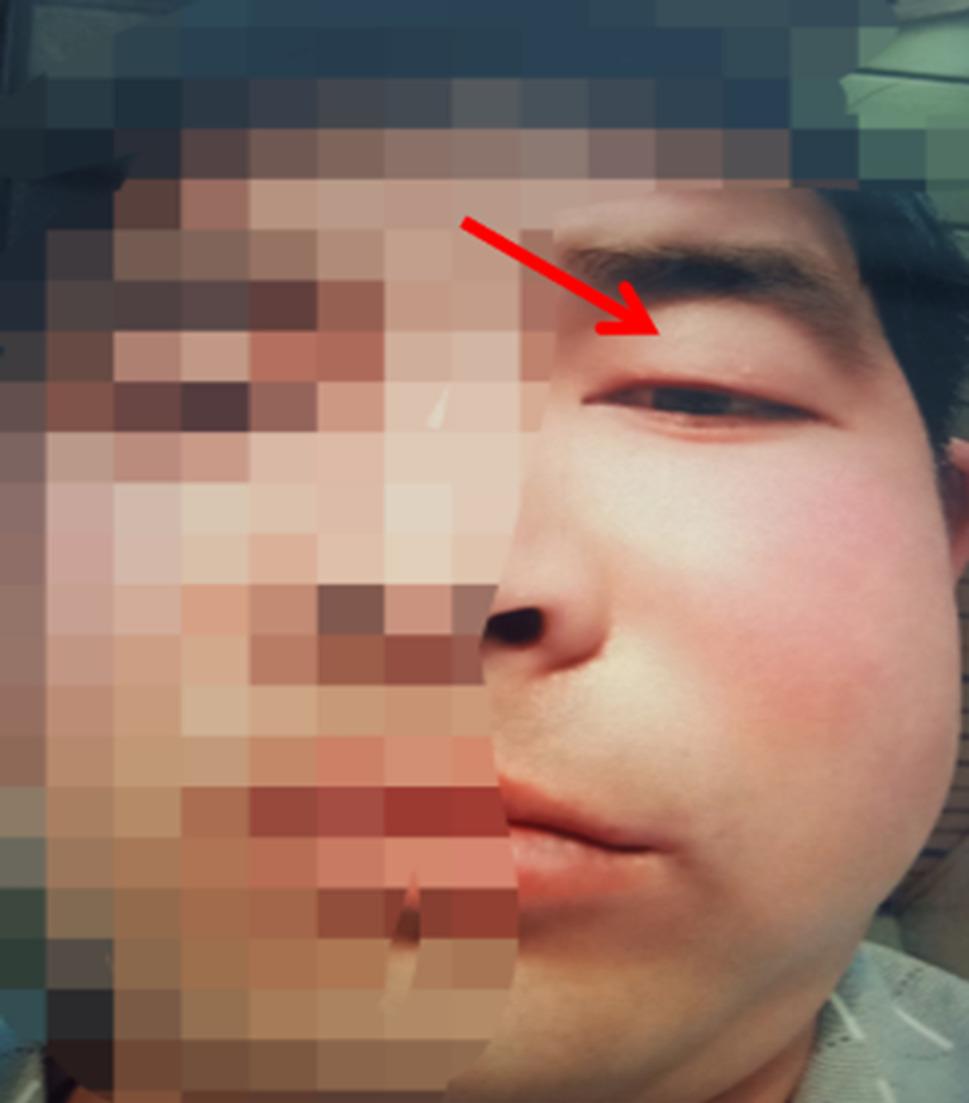
Preoperative clinical presentation. A firm, palpable mass in the left maxillary region accompanied by severe periorbital edema, causing mechanical ptosis and preventing spontaneous opening of the left eye

**Fig. 2:**
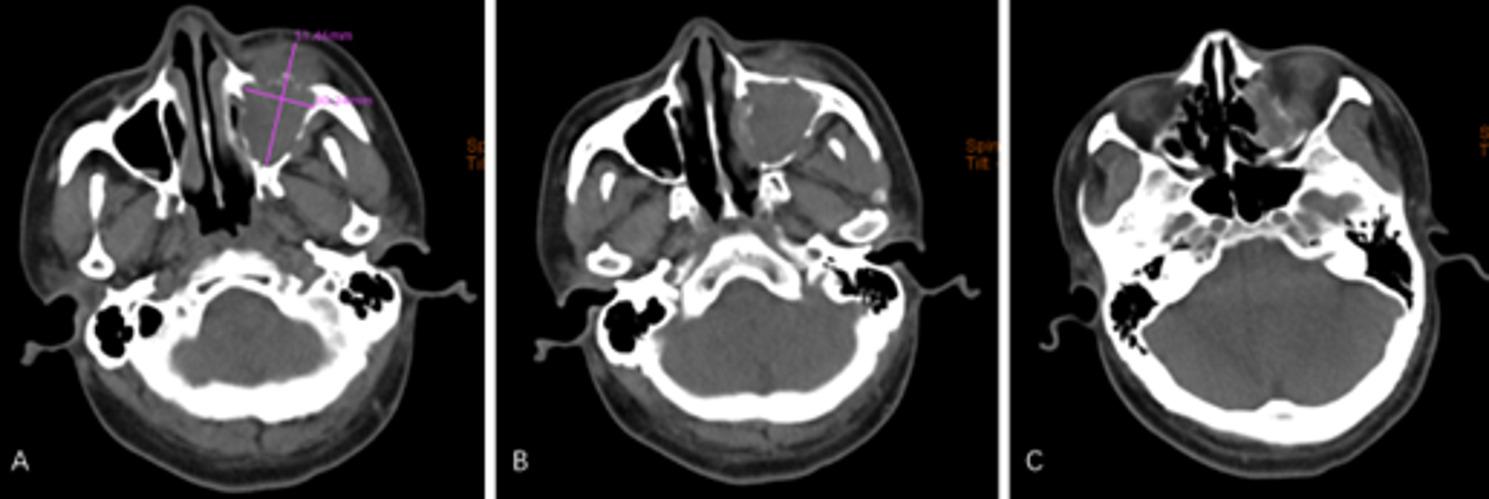
February 3, 2021. **A **An irregular mass (5.1 cm × 3.0 cm) in the left maxillofacial region. **B** Soft tissue swelling with internal nodular calcifications, opacification of the maxillary and ethmoid sinuses, and extensive osteolytic destruction of the sinus walls. **C** Tumor extension into the subcutaneous soft tissue and compression of the optic nerve

**Fig. 3:**
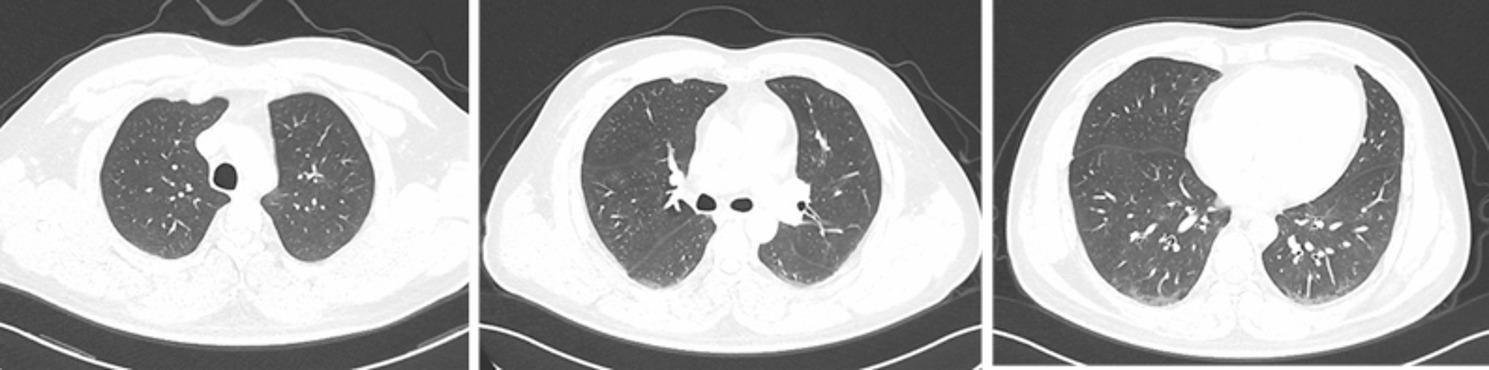
February 3, 2021

On February 3, 2021, a 37-year-old male presented to the Department of Oral and Maxillofacial Surgery with a one-month history of left facial swelling and pain, accompanied by an acute exacerbation of intractable pain and visual impairment over the preceding two days. The patient had no significant past medical history, and his family history was negative for genetic disorders or malignancies. Physical examination revealed a firm, fixed palpable mass in the left maxillary region, with intact overlying skin. Severe signs of orbital involvement were evident, including marked eyelid and periorbital edema that prevented spontaneous eye opening (Fig. 1). Subjectively, the patient reported diplopia and significant blurring of vision. Emergency CT revealed an irregular soft tissue mass (approximately 5.1 cm × 3.0 cm) in the left maxillary region with extensive osteolytic destruction of the anterior, medial, and lateral walls of the maxillary sinus; the tumor extended into the subcutaneous soft tissue and compressed the optic nerve (Fig. 2). Additionally, chest CT revealed no obvious abnormalities (Fig. 3).

**Fig. 4:**
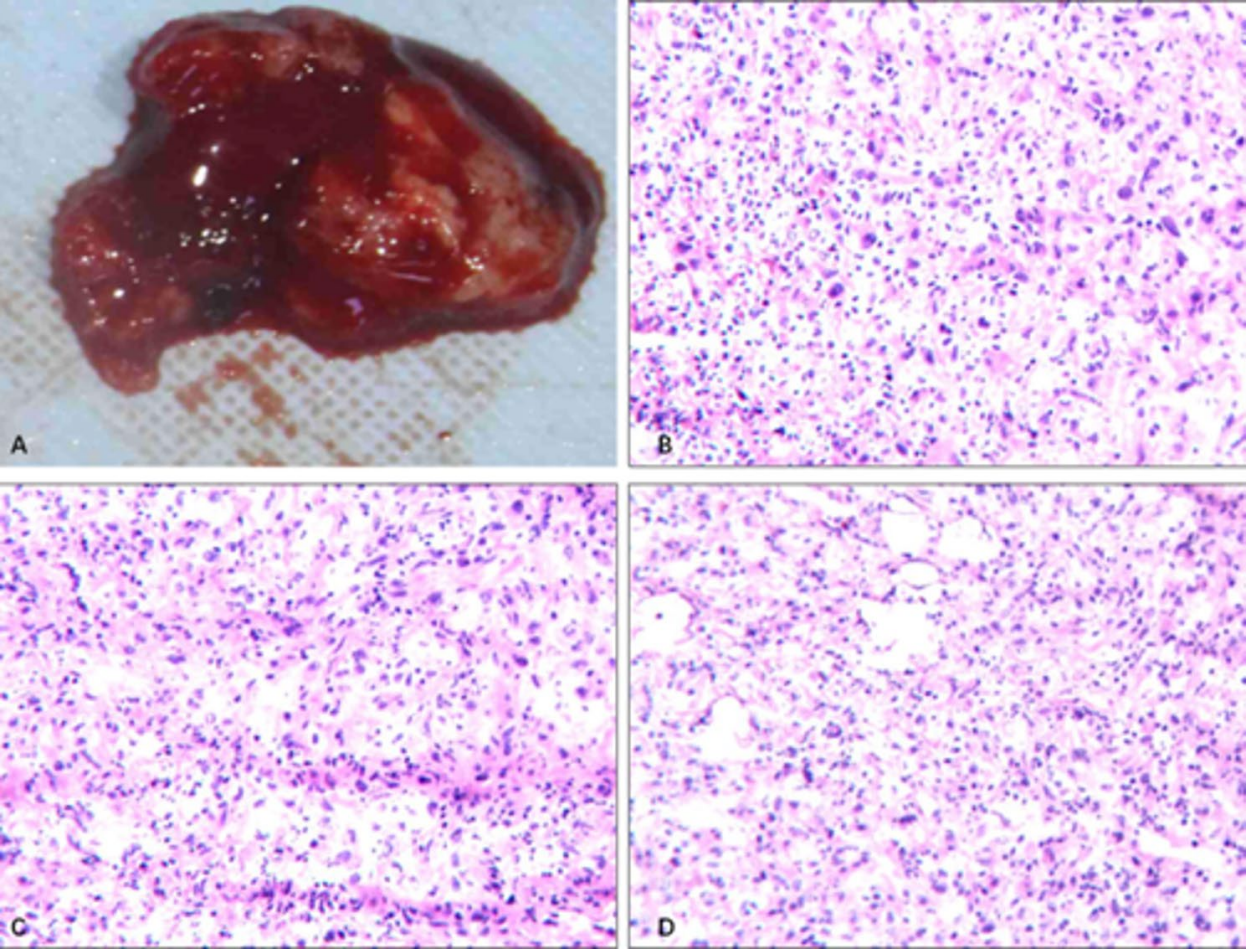
Intraoperative gross specimen and rapid frozen section pathology. **A** Gross specimen of the resected tumor. **B-D** Intraoperative rapid frozen section pathology (H&E staining) reveals a high-grade spindle cell mesenchymal neoplasm

**Fig. 5:**
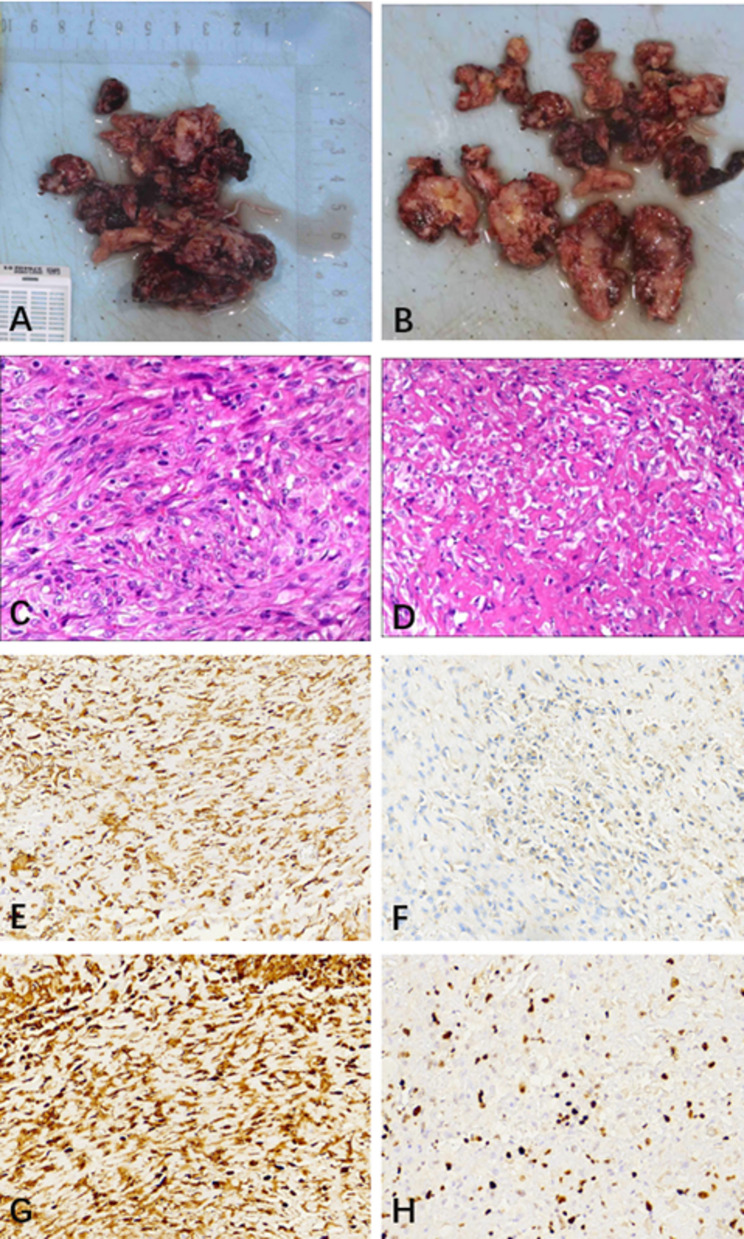
Postoperative histopathology and immunohistochemistry. **A**, **B** Gross specimen. **C**, **D** **H**&**E** staining revealing neoplastic cells with spindle and polygonal phenotypes, characterized by marked cellular atypia, occasional mitotic figures, and the presence of eosinophilic tumor osteoid matrix. Immunohistochemical staining showing: **E** Partial positivity for SMA; **F** Scattered positivity for CD68; **G** Positivity for CKP; **H** A high Ki-67 proliferation index of approximately 70%

We acknowledge that proceeding directly to surgery without definitive preoperative histopathology deviated from standard oncologic principles. However, given the immediate threat of permanent blindness and severe pain, the patient underwent an emergency subtotal maxillectomy on February 4, 2021, as a compassionate, vision-preserving measure. During the procedure, an intraoperative frozen section identified a “spindle cell mesenchymal neoplasm” (Fig. 4). Although the definitive subtype remained indeterminate at that moment, the confirmation of malignancy—compounded by the critical need for orbital decompression—dictated the surgical strategy. A standard en-bloc resection was deemed technically unfeasible without sacrificing visual function due to tumor friability and adhesions. Consequently, a palliative piecemeal resection was performed (Fig. 5A-B). The resulting defect was packed with iodoform gauze via the inferior meatus. In retrospect, it is acknowledged that this suboptimal resection likely resulted in microscopic residual disease, which is identified as the precipitating factor for the rapid local recurrence observed one month postoperatively.

Postoperative immunohistochemical (IHC) analysis revealed that the tumor cells were positive for CKP and Vimentin, partially positive for SMA, and showed scattered positivity for CD68. Conversely, markers such as Desmin, S-100, CD34, HMB45, and SOX-10 were negative. The Ki-67 proliferation index was 70%, and p53 showed a wild-type expression pattern (80%) (Fig. 5C-H). The negativity for Desmin, S-100, and CD34 effectively excluded rhabdomyosarcoma, neurogenic tumors, and vascular neoplasms [16]. Although CKP positivity is uncommon in conventional osteosarcoma, aberrant epithelial marker expression is a documented phenomenon in high-grade variants [17, 18]. Crucially, the definitive diagnosis was confirmed by the presence of malignant osteoid matrix produced directly by tumor cells on H&E staining. Consequently, the lesion was diagnosed as Osteosarcoma of the left maxilla, classified as Enneking Stage IIB (High-grade, Extracompartmental).

**Fig. 6:**
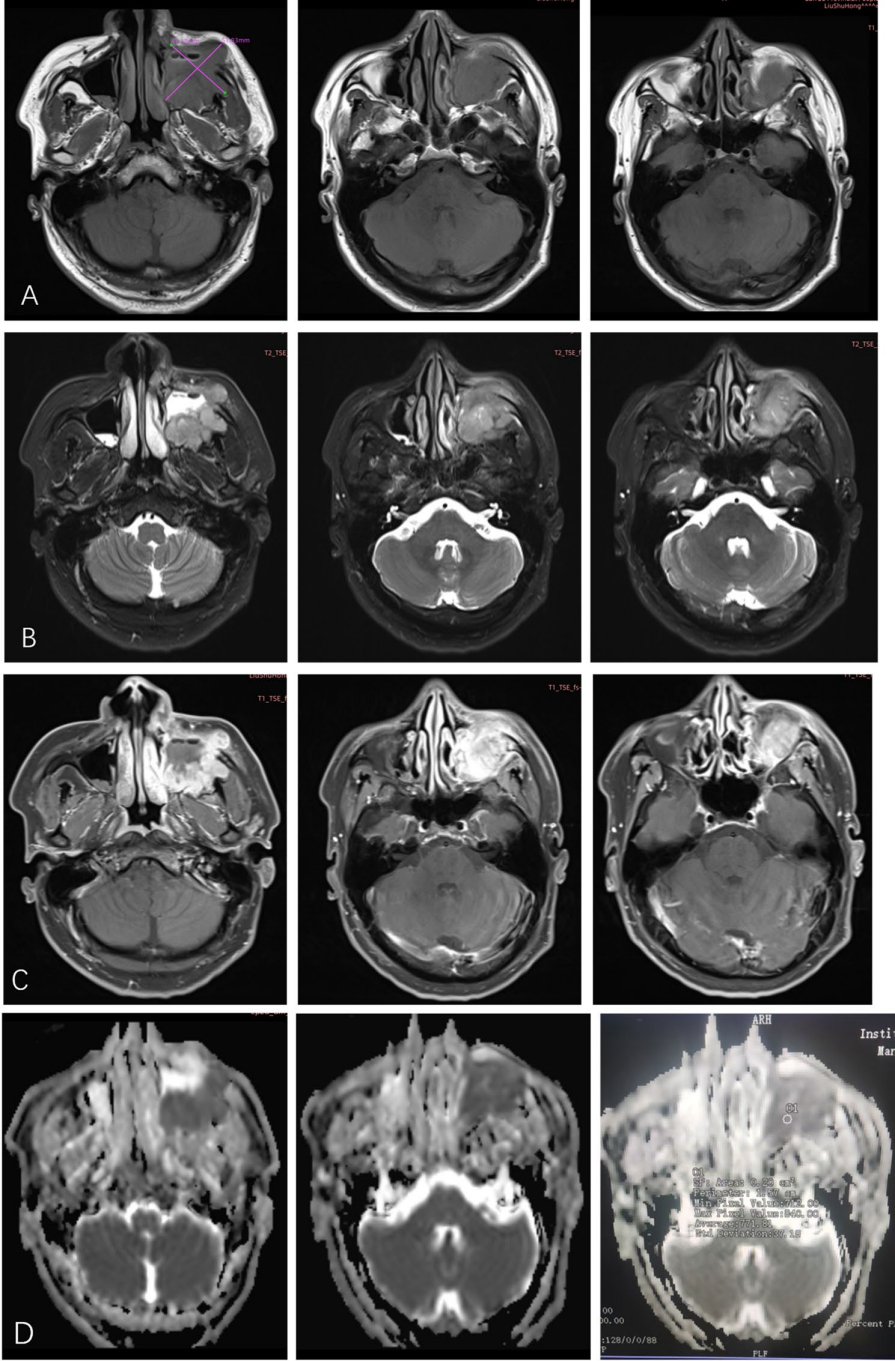
Enhanced Maxillofacial MRI from March 8, 2021, demonstrating local recurrence. **A** T1-weighted image showing an irregular hypointense mass (5.2 cm × 4.7 cm) in the left maxilla. **B** T2-weighted image showing soft tissue swelling with indistinct margins blurring the boundary with the ethmoid sinus. **C** Contrast-enhanced T1-weighted image showing marked heterogeneous enhancement filling the sinus and infiltrating the subcutaneous fat. **D** ADC showing low signal intensity within the mass, confirming restricted diffusion characteristic of tumor recurrence

On March 3, 2021, the patient presented to the Department of Radiation Oncology with a recurrence of left maxillofacial swelling and diplopia. Contrast-enhanced MRI revealed a heterogeneously enhancing mass with ill-defined borders in the left maxillary region, extending into the ethmoid sinus and posterior to the optic nerve (Fig. 6A-C). To differentiate tumor recurrence from postoperative inflammatory changes or edema, quantitative analysis of Apparent Diffusion Coefficient (ADC) maps was performed. The lesion demonstrated distinct restricted diffusion with a mean ADC value of 0.77 × 10⁻³ mm²/s (Fig. 6D).

This quantitative finding serves as critical diagnostic evidence, as supported by the following literature: (1) Wang et al. [19]reported that the mean ADC value for malignant bone tumors is 0.87 ± 0.20 × 10⁻³ mm²/s, significantly lower than that of benign lesions (1.17 ± 0.36 × 10⁻³ mm²/s). They established an ADC cut-off value of 1.10 × 10⁻³ mm²/s for differentiating malignancy with a sensitivity of 89.7% and specificity of 84.5%. (2) Adding functional sequences (DWI/ADC) to conventional MRI increases the specificity for detecting recurrence from 52% to 97%. The recurrence (mean ADC 1.08 ± 0.19 × 10⁻³ mm²/s) is distinct from those of hematomas (mean ADC 2.34 ± 0.72 × 10⁻³ mm²/s) and postoperative scarring (mean ADC 0.9 ± 0.00 × 10⁻³ mm²/s) [20]. (3) Viable tumor areas exhibit low ADC values (mean 0.8 ± 0.3 × 10⁻³ mm²/s), whereas necrotic areas show significantly higher values (mean 2.3 ± 0.2 × 10⁻³ mm²/s) due to increased water diffusivity [21]. In summary, our patient’s measured value (0.77 × 10⁻³ mm²/s) falls strictly within the ranges for viable malignancy established by these studies, supporting the diagnosis. In our case, the rapid interval growth (measuring approximately 5.2 cm × 4.7 cm within just one month), combined with distinct restricted diffusion and convex margins, supported a diagnosis of solid malignancy rather than postoperative changes. The diagnosis of recurrence was thus established as a clinicoradiological assessment.

We explicitly acknowledge that the lack of histopathological confirmation is a limitation. However, this decision reflected a necessary balance between academic rigor and patient safety. Given the lesion’s proximity to the skull base and compression of the optic nerve, re-biopsy carried significant risks of hemorrhage and iatrogenic neural injury [22–24]. Since the initial emergency surgery was undertaken specifically to preserve vision, the patient refused to accept the risk of a biopsy that threatened blindness, as this would have negated the primary therapeutic objective. Therefore, based on the high confidence of functional MRI and respect for the patient’s strong preference, we proceeded directly to salvage therapy.

Given the highly aggressive nature of the recurrence and the significant tumor burden, an intensified multimodal salvage protocol was adopted. On March 5, 2021, the patient received the first cycle of chemotherapy comprising Epirubicin (100 mg, IV, d1, q21d) and Cisplatin (120 mg, IV, d1, q21d), combined with targeted therapy using Anlotinib (12 mg, orally, qd, d1-14, q21d). This regimen represents a variation of the standard Doxorubicin and Cisplatin (AP) protocol. It was selected in accordance with the NCCN Guidelines for Bone Cancer (Version 1.2021), which was the active standard of care at the time of treatment [25]. Although the disease was recurrent, this first-line protocol was selected because the patient was chemotherapy-naïve, having not received neoadjuvant or adjuvant therapy initially. Epirubicin was selected as the anthracycline component to mitigate potential cardiotoxicity while maintaining comparable antitumor efficacy to Doxorubicin, a strategy supported by systematic reviews [26]. Regarding dosage, standard full-dose chemotherapy carries high toxicity risks when combined with radical radiotherapy and targeted therapy. To ensure safety and treatment completion, we applied a proactive dose reduction of approximately 20–30% tailored to the patient’s body surface area (1.77 m²). This individualized strategy aimed to balance antitumor efficacy with the cumulative toxicity of the triple-modality regimen.

**Fig. 7:**
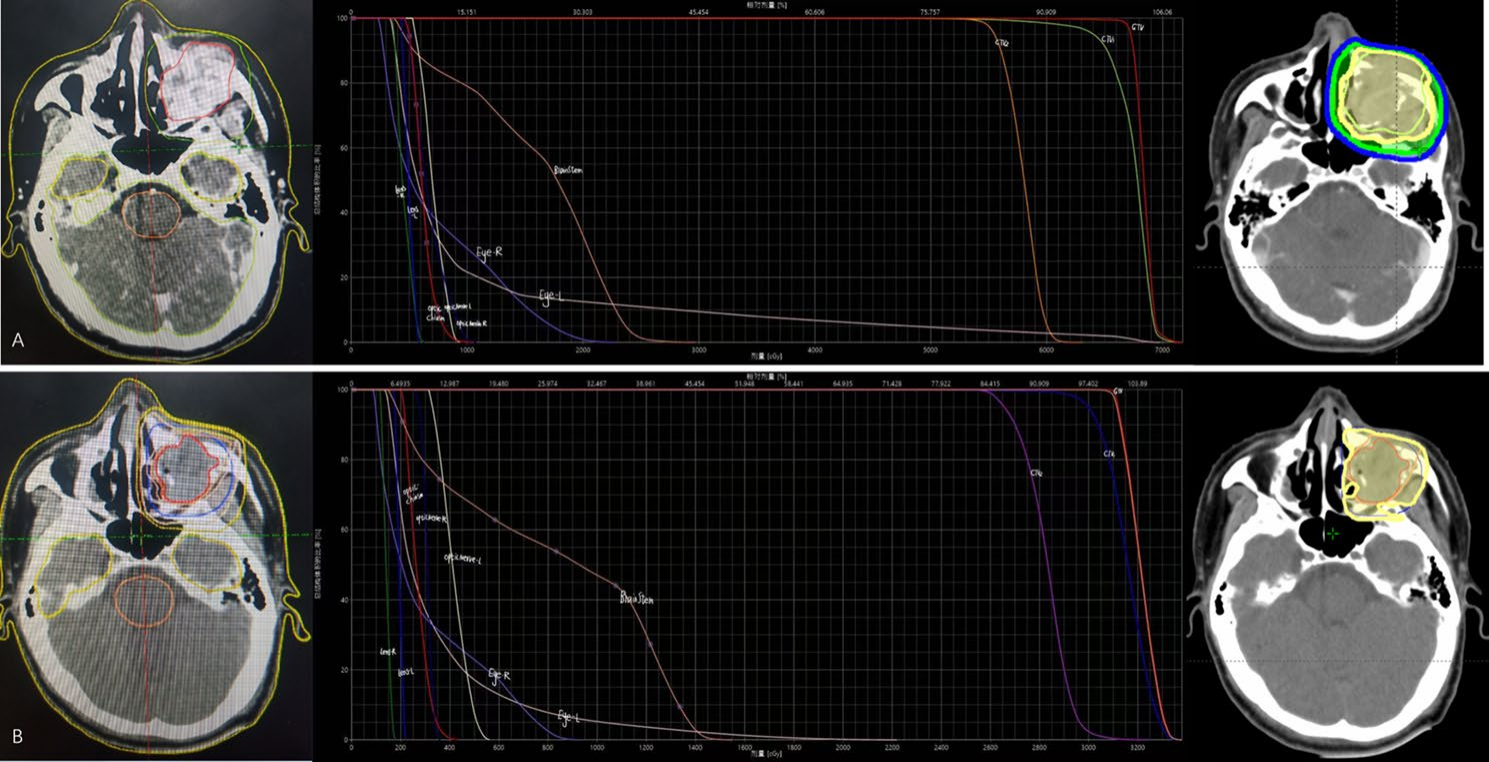
Radiotherapy planning. **A** Initial plan (March 8, 2021) displaying the Gross Tumor Volume (GTV) and Dose-Volume Histogram (DVH). **B** Adaptive plan with field reduction (March 29, 2021) implemented after 15 fractions to spare the optic nerve, showing the revised target volume

Subsequently, concurrent intensity-modulated radiotherapy (IMRT) was administered from March 9 to April 22, 2021, delivering a total dose of 66 Gy in 30 fractions. This dose intensity was selected in accordance with NCCN Guidelines for Bone Cancer, which recommend cumulative radiation doses in the range of 60–70 Gy for unresectable or macroscopically residual high-grade sarcomas to maximize the probability of local control in this typically radio-resistant tumor [25]. Our dose of 66 Gy falls precisely within these recommended ranges to maximize local control while respecting the tolerance of critical head and neck structures. Notably, after 15 fractions, the facial mass showed marked regression, prompting a re-planning with field reduction to better spare the optic nerve, globe, and other critical structures (Fig. 7).

**Fig. 8:**
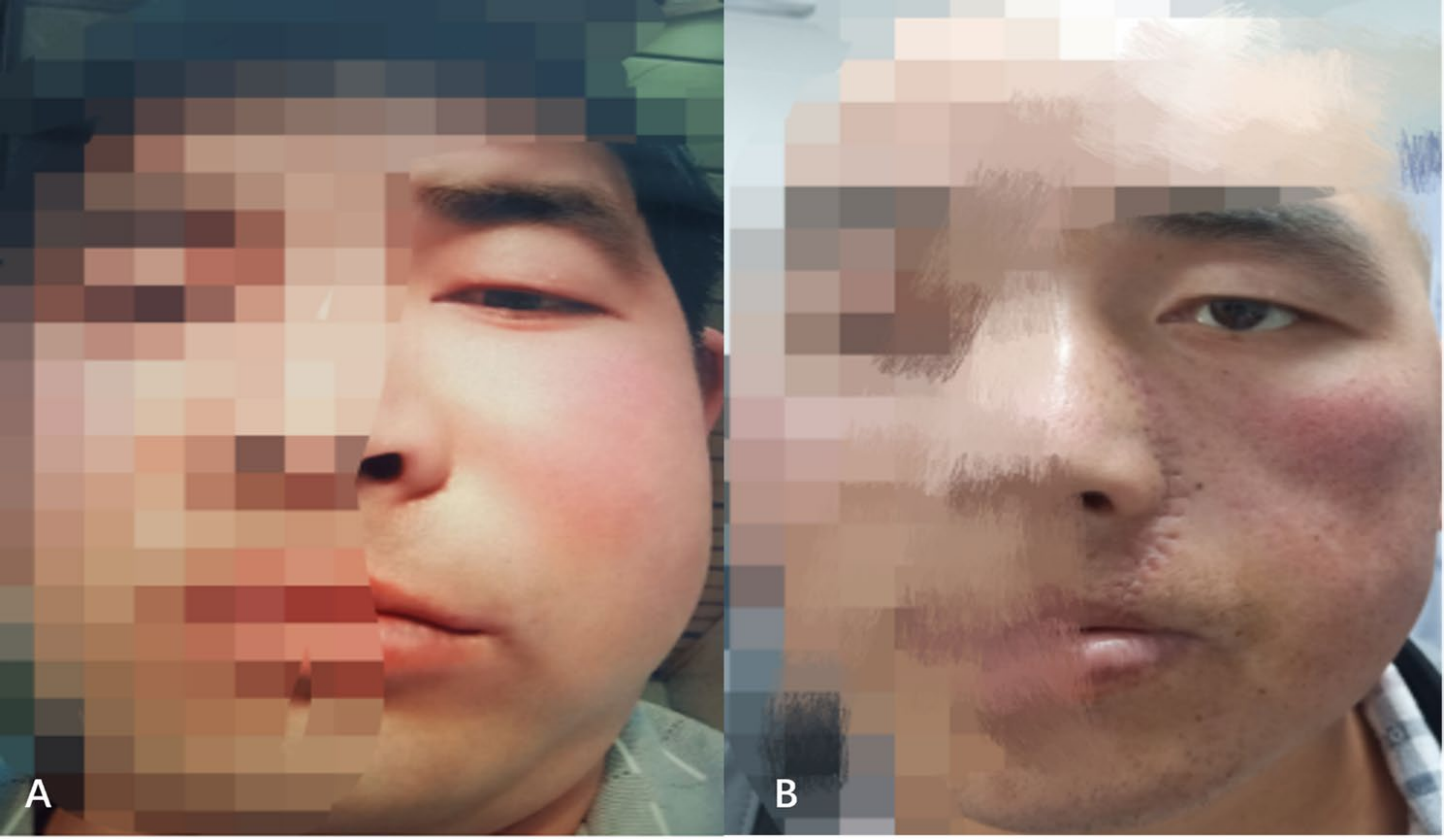
Clinical response to treatment. **A** Pre-treatment presentation (February 3, 2021) with severe edema and mechanical ptosis. **B** Post-treatment presentation (April 23, 2021) showing significant resolution of swelling and restoration of the ability to open the left eye

**Fig. 9:**
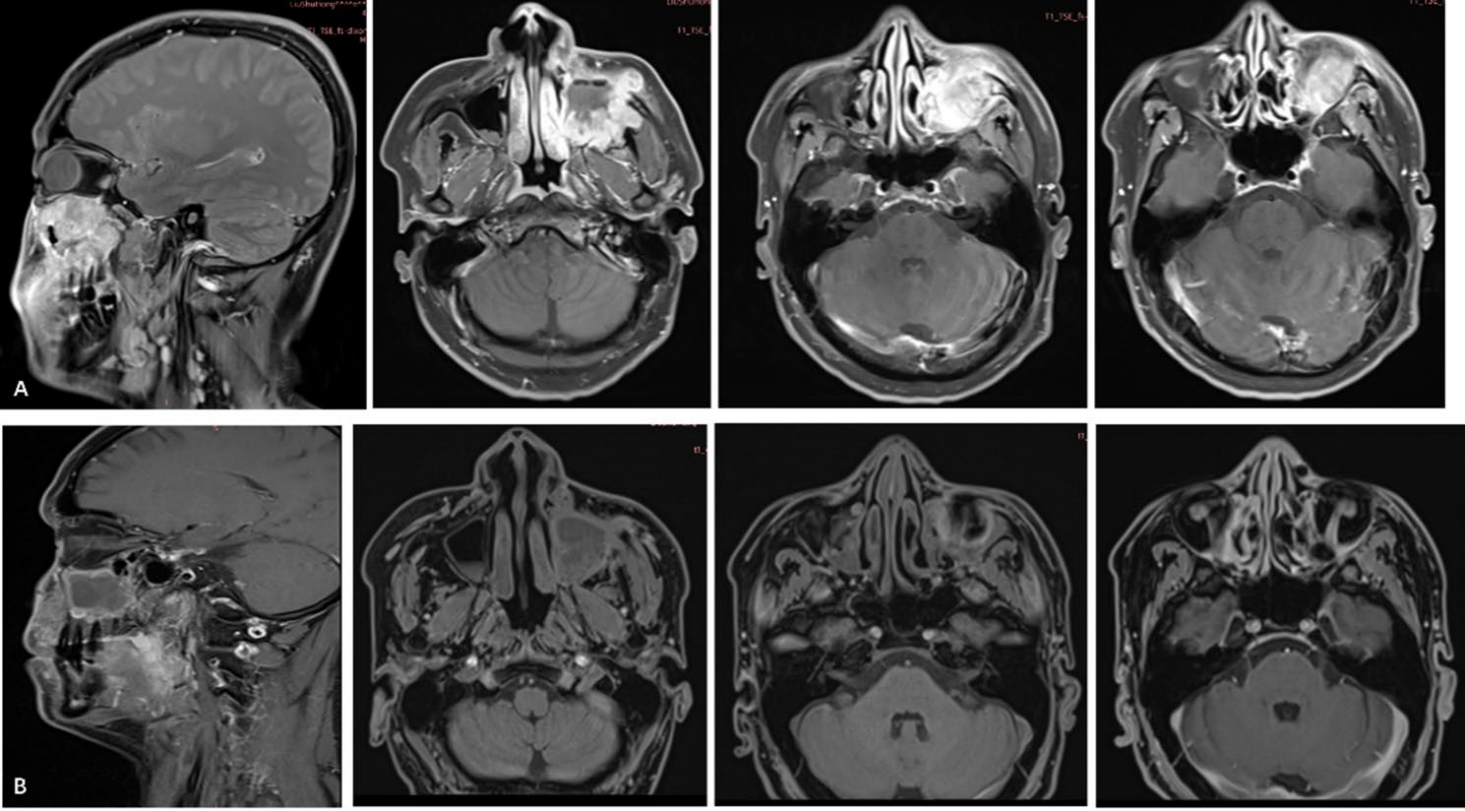
Radiological response assessment. **A** MRI (March 8, 2021) showing tumor compression of the optic nerve. **B** MRI (May 19, 2021) revealing a clear fat plane between the residual lesion and the optic nerve

On April 21, 2021, the patient received the second cycle of the same chemotherapy and Anlotinib regimen. By the conclusion of radiotherapy, the facial mass had regressed significantly. Clinically, the severe periorbital edema resolved, allowing the patient to open the left eye spontaneously (Fig. 8). The patient reported the complete resolution of diplopia and blurred vision. Although formal visual acuity testing was not performed, this functional symptomatic improvement was consistent with the MRI, which confirmed the decompression of orbital structures and the regression of the tumor away from the optic nerve (Fig. 9).

Following the completion of radiotherapy, the patient underwent four additional cycles of consolidation therapy with the same chemotherapy and Anlotinib regimen on May 18, June 10, July 13, and August 17, 2021. After the final cycle, Anlotinib monotherapy was continued as maintenance. Throughout the multimodal treatment course, the regimen was well-tolerated. Adverse events were limited to Grade I myelosuppression and mild hand-foot syndrome (attributed to Anlotinib), both of which were manageable with symptomatic care and did not necessitate treatment discontinuation.

**Fig. 10:**
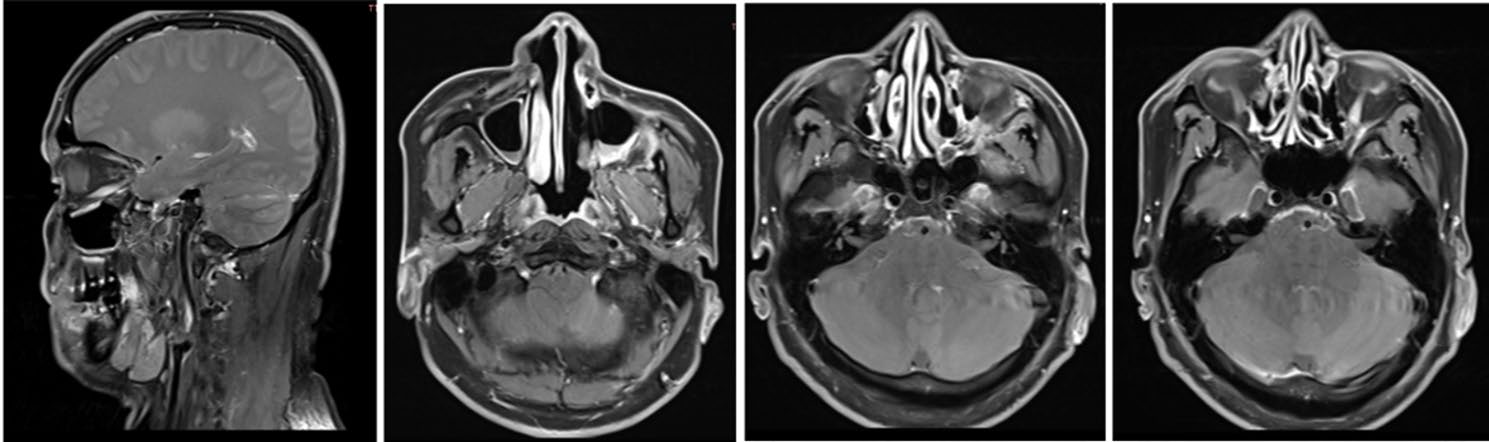
February 17, 2025. The imaging demonstrates no evidence of local recurrence

**Fig. 11:**
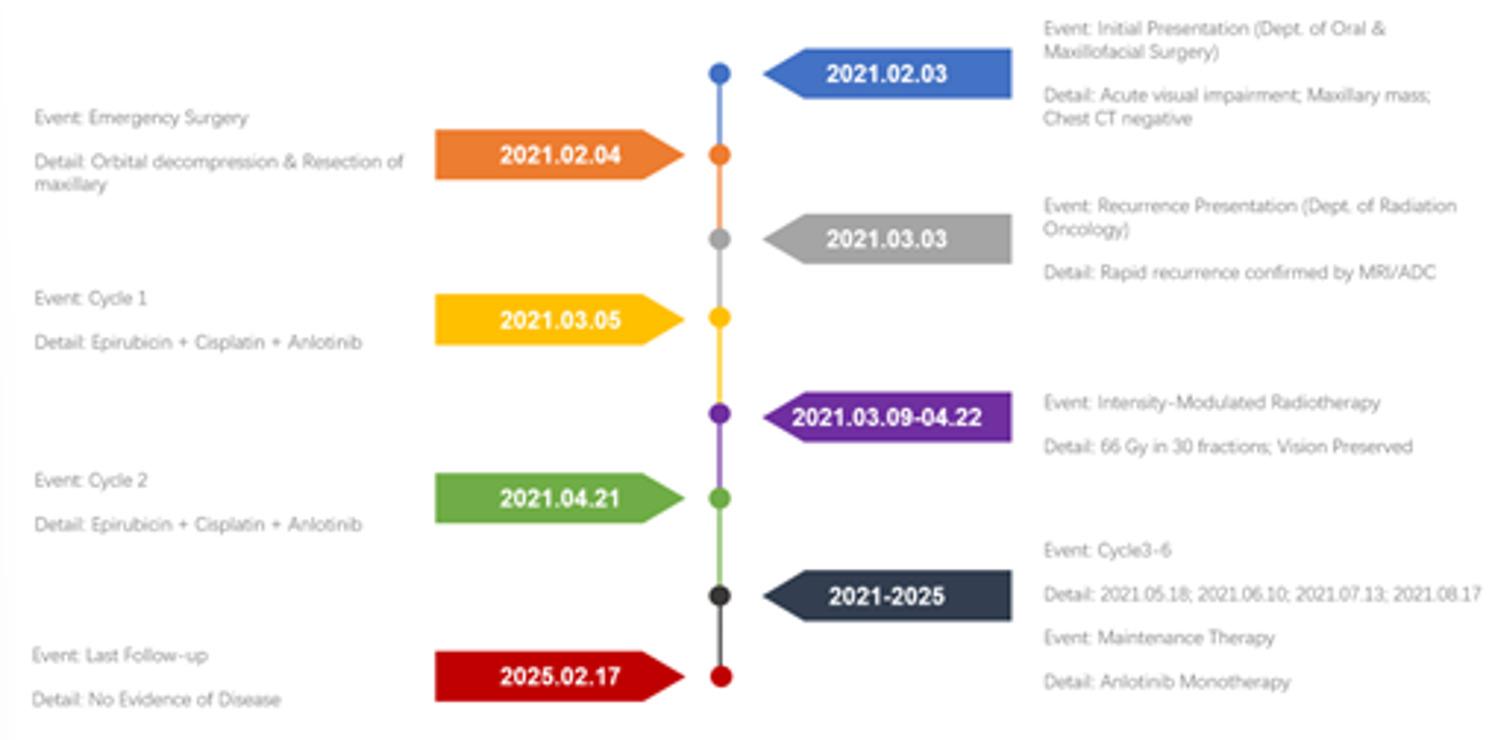
Timeline of Clinical Management

Regular surveillance with cranial MRI and thoracoabdominal CT was performed. As of February 17, 2025, the patient maintains No Evidence of Disease (NED) status based on clinical and radiological follow-up (Fig. 10). A comprehensive timeline of the patient’s diagnosis, treatment, and follow-up is illustrated in Fig. 11.

## Patient perspective

When the tumor grew back so quickly after the first surgery, I was terrified. I thought I would lose my eye and my life. The doctors explained the risks of the new treatment, but I had no other choice. During the treatment, my vision improved day by day, which gave me hope. Although the side effects were tiring, being able to see my family and live a normal life four years later is a miracle to me. I am grateful we took the aggressive approach.

## Discussion

JOS exhibits distinct epidemiological and biological characteristics compared to conventional extremity osteosarcoma. A unique therapeutic challenge in JOS is the complex anatomy of the midface, which often precludes wide surgical margins—the most critical prognostic factor for local control [5, 6, 27, 28].

A critical point of discussion in this case involves the initial non-standard surgical management performed prior to the patient’s referral to our Department of Radiation Oncology. We explicitly acknowledge that proceeding to surgery without definitive preoperative histopathological subtyping deviated from standard osteosarcoma management principles. We do not endorse this approach for routine practice and strongly advise strict adherence to preoperative biopsy protocols whenever feasible. However, this “shortcut” was driven by the emergency nature of the orbital compression. An intraoperative frozen section confirmed the presence of a spindle cell mesenchymal neoplasm, and the imminent threat of permanent blindness necessitated emergency intervention. Interestingly, recent large-scale data challenges the absolute necessity of the standard neoadjuvant chemotherapy approach. A study analyzing SEER data (2007–2019) found that upfront surgery followed by adjuvant chemotherapy yielded a 5-year survival of 74%, compared to 67% in the neoadjuvant-first group [29]. This suggests that in specific contexts, immediate tumor removal to reduce burden is a valid strategy. Therefore, while our decision was driven by the ocular emergency, it may inadvertently have aligned with emerging evidence favoring upfront surgery.

We acknowledge that histopathological confirmation of recurrence is the gold standard and advocate for strict adherence to this principle in clinical practice. However, the patient’s initial emergency surgery was undertaken specifically to preserve the optic nerve, highlighting the immense value he placed on his vision and quality of life. Following recurrence, the patient refused to accept the risk of a biopsy that threatened iatrogenic blindness, as this would have negated the primary objective of the previous treatment. In this context, functional MRI provided critical diagnostic support. Del Grande et al. [20] demonstrated that while conventional contrast-enhanced MRI has a specificity of only 52% for differentiating recurrence from scarring, the addition of DWI and ADC mapping increases specificity to 97%. Furthermore, Wang et al. [19] established an ADC cut-off value of 1.10 × 10⁻³ mm²/s for differentiating malignancy with high sensitivity. In our case, based on the specific ADC value (0.77 × 10⁻³ mm²/s) and rapid interval growth, we diagnosed recurrence clinicoradiologically and initiated salvage therapy.

Previous reports on jaw osteosarcoma salvage primarily emphasize the role of radical re-resection. This case is distinct because it provides evidence for a non-surgical salvage pathway. Data indicates that for patients with positive margins, radiotherapy may improve Overall Survival from 31% to 80% [6]. To further enhance control, the decision to incorporate Anlotinib was based on clinical guidelines and emerging mechanistic evidence. Tyrosine Kinase Inhibitors (TKIs) have been recommended by the NCCN guidelines as second-line therapy for advanced osteosarcoma that progresses after chemotherapy [30]. Specifically in China, Anlotinib has been designated as a Class I recommendation by the CSCO guidelines for advanced bone and soft tissue sarcomas [9]. Given the patient’s rapid progression, high tumor burden, and contraindication for re-resection, we adopted an aggressive multimodal therapeutic regimen.

The retrospective review by Tian et al. [11] documented the efficacy and manageable safety profile of Anlotinib. Furthermore, Li et al. [31] reported a median Progression-Free Survival (PFS) of 9.8 months in advanced osteosarcoma, supporting its robust clinical utility. Importantly, a Phase II clinical trial (NCT03527888) reported an encouraging Disease Control Rate (DCR) of 78.57% in refractory bone tumors, with a median PFS of 4.83 months specifically for the osteosarcoma subgroup [13].

Additionally, studies suggest Anlotinib not only suppresses osteosarcoma growth via dual blockade of VEGFR2 and MET but also enhances the sensitivity of osteosarcoma cells to Cisplatin by promoting apoptosis [10]. Radiotherapy can promote the exposure of tumor tissue antigens, and when combined with Anlotinib, may enhance the Anlotinib-induced immune response, further contributing to disease control [32]. These findings provided a strong molecular rationale for our concurrent administration. Given the rapidity of the recurrence, sustained control was critical. Data indicates that “switch maintenance therapy” with Anlotinib in unresectable sarcoma yields a DCR of 81.0% [14], supporting our strategy to prevent a second recurrence. Collectively, these findings provided a robust rationale for our triple-modality regimen.

However, due to the limitations inherent to single-case reports, isolating the specific contribution of Anlotinib within this intensive triple-modality regimen is challenging. It is also crucial to emphasize that unlike targeted therapies requiring specific mutation screening (e.g., EGFR inhibitors in lung cancer), Anlotinib targets ubiquitous angiogenic pathways (VEGFR, FGFR, PDGFR) that are universally upregulated in osteosarcoma [15]. Pivotal clinical trials validating Anlotinib in sarcoma and specific studies on osteosarcoma, did not require biomarker selection for enrollment [33–36]. Therefore, despite the lack of specific pathway analysis, our empirical use of this agent was consistent with CSCO guidelines and standard clinical practice. To our knowledge, this is one of the few reports documenting the long-term success of this specific multimodal combination for recurrent JOS.

## Limitations

This study has several limitations inherent to single-case reports and the specific clinical circumstances. (1) The findings reflect the experience of a single patient, and generalized efficacy cannot be established without validation in larger-scale prospective trials. (2) The diagnosis of recurrence was established based on clinicoradiological criteria (quantitative ADC/MRI) without biopsy. We explicitly acknowledge the absence of histopathological confirmation as a significant limitation. However, given the critical anatomical location, the risks of iatrogenic blindness and severe hemorrhage outweighed the benefit of confirmation. Crucially, this decision respected the patient’s strong preference: since the initial emergency surgery was undertaken specifically to preserve visual function, performing a high-risk biopsy that threatened vision would have negated the primary therapeutic objective of the original intervention. (3) Several critical deviations from standard oncologic guidelines occurred due to the emergency presentation. These included: (a) proceeding to surgery without definitive preoperative histopathology; and (b) omission of standard neoadjuvant chemotherapy. We emphasize that oncologic resection is typically not an emergency procedure and should ideally be planned within a Multidisciplinary Team. These deviations, while driven by the immediate threat of blindness, undoubtedly complicated the clinical course and the interpretation of outcomes. 4) No patient-specific genetic sequencing was performed to identify specific targets or mutations. However, as discussed, Anlotinib targets broad angiogenic pathways universally upregulated in osteosarcoma, and guidelines do not mandate such testing for its empirical use. 5) The visual recovery was patient-reported and lacks quantitative ophthalmologic data or formal ophthalmologic consultation records. 6) Given the multimodal nature of the salvage therapy (concurrent chemotherapy, radiotherapy, and TKI), isolating the specific contribution of Anlotinib remains challenging.

## Conclusion

In conclusion, this case highlights the formidable challenges of managing osteosarcoma following unavoidable compromises in initial surgical management. While the emergency intervention successfully preserved vision, the omission of standard neoadjuvant therapy and en-bloc resection likely precipitated rapid recurrence. Furthermore, the decision to forego biopsy at recurrence—driven by the young patient’s prioritization of vision and quality of life, alongside strong personal preference—undoubtedly complicated clinical decision-making. We reiterate that we do not endorse deviating from standard oncologic principles. However, the primary significance of this report is to demonstrate that in refractory cases where salvage surgery is contraindicated, an aggressive multimodal strategy—integrating IMRT, systemic chemotherapy, and Anlotinib—has the potential to achieve durable clinical remission. Supported by clinical guidelines and evidence of therapeutic synergy, these findings suggest a potential role for integrating TKIs in complex salvage settings. These observations should be interpreted with caution and warrant validation in large-scale, prospective randomized trials.

## Data Availability

All data generated or analysed during this study are included in this published article. To protect the patient’s privacy, the original medical records and imaging data are not publicly available but are available from the corresponding author upon reasonable request.

## Abbreviations

OS: Osteosarcoma
JOS: Osteosarcoma of the jaw
CSCO: Chinese Society of Clinical Oncology
IHC: immunohistochemical
ADC: Apparent Diffusion Coefficient
AP: Doxorubicin and Cisplatin
IMRT: intensity-modulated radiotherapy
NED: No Evidence of Disease
TKIs: tyrosine kinase inhibitors
DCR: disease control rate
PFS: progression-free survival
GTV: gross tumor volume
DVH: Dose and Volume Histogram

## References

[1] Wang X, Zhu K, Hu J, Zhang C. Advances and challenges in the treatment of osteosarcoma. Prog Biophys Mol Biol. 2025;197:60–74. 40618789 10.1016/j.pbiomolbio.2025.07.001

[2] Picci P. Osteosarcoma (osteogenic sarcoma). Orphanet J Rare Dis. 2007;2:6. 17244349 10.1186/1750-1172-2-6 PMC1794406

[3] Rodriguez-Molinero J, Pozo-Kreilinger JJ, Ruiz-Roca JA, Lopez-Sanchez AF, Cebrian-Carretero JL. Clinical and Pathological Features of Osteosarcomas of the Jaws: A Retrospective Study. Clin Pract. 2024;14(3):965–79. 38921255 10.3390/clinpract14030077 PMC11202223

[4] Lee RJ, Arshi A, Schwartz HC, Christensen RE. Characteristics and prognostic factors of osteosarcoma of the jaws: a retrospective cohort study. JAMA Otolaryngol Head Neck Surg. 2015;141(5):470–7. 25811167 10.1001/jamaoto.2015.0340

[5] Patel SG, Meyers P, Huvos AG, Wolden S, Singh B, Shaha AR, Boyle JO, Pfister D, Shah JP, Kraus DH. Improved outcomes in patients with osteogenic sarcoma of the head and neck. Cancer. 2002;95(7):1495–503. 12237918 10.1002/cncr.10849

[6] Guadagnolo BA, Zagars GK, Raymond AK, Benjamin RS, Sturgis EM. Osteosarcoma of the jaw/craniofacial region: outcomes after multimodality treatment. Cancer. 2009;115(14):3262–70. 19382187 10.1002/cncr.24297

[7] Zhang L, Guo Y, Sumita Y, Hirai H, Yamazaki M, Hayashi T, et al. Does chemotherapy improve the prognosis in patients with jaw osteosarcoma? A systematic review and meta-analysis. Int J Oral Maxillofac Surg. 2026;55(1):10–19. 10.1016/j.ijom.2025.09.008 Epub 2025 Sep 29. PMID: 41027777. 10.1016/j.ijom.2025.09.008 41027777

[8] Baumhoer D, Brunner P, Eppenberger-Castori S, Smida J, Nathrath M, Jundt G. Osteosarcomas of the jaws differ from their peripheral counterparts and require a distinct treatment approach. Experiences from the DOESAK Registry. Oral Oncol. 2014;50(2):147–53. 24246156 10.1016/j.oraloncology.2013.10.017

[9] Chen Q, Zheng K, Xu M, Yan N, Hai G, Yu X. Anlotinib combined with radiotherapy and chemotherapy for recurrent pelvic osteosarcoma treatment: a case report and literature review. Front Oncol. 2023;13:1283932. 38156107 10.3389/fonc.2023.1283932 PMC10753991

[10] Wang G, Sun M, Jiang Y, Zhang T, Sun W, Wang H, Yin F, Wang Z, Sang W, Xu J, et al. Anlotinib, a novel small molecular tyrosine kinase inhibitor, suppresses growth and metastasis via dual blockade of VEGFR2 and MET in osteosarcoma. Int J Cancer. 2019;145(4):979–93. 30719715 10.1002/ijc.32180

[11] Tian Z, Liu H, Zhang F, Li L, Du X, Li C, Yang J, Wang J. Retrospective review of the activity and safety of apatinib and anlotinib in patients with advanced osteosarcoma and soft tissue sarcoma. Invest New Drugs. 2020;38(5):1559–69. 32100146 10.1007/s10637-020-00912-7 PMC7497688

[12] Sun Y, Niu W, Du F, Du C, Li S, Wang J, Li L, Wang F, Hao Y, Li C, et al. Safety, pharmacokinetics, and antitumor properties of anlotinib, an oral multi-target tyrosine kinase inhibitor, in patients with advanced refractory solid tumors. J Hematol Oncol. 2016;9(1):105. 27716285 10.1186/s13045-016-0332-8 PMC5051080

[13] Tang L, Niu X, Wang Z, Cai Q, Tu C, Fan Z, Yao Y. A phase II study of anlotinib in treating patients with relapsed or metastatic primary malignant bone tumor. J Clin Oncol. 2020;38(15suppl):11525–11525.

[14] Liu J, Deng YT, Jiang Y. Switch maintenance therapy with anlotinib after chemotherapy in unresectable or metastatic soft tissue sarcoma: a single-center retrospective study. Invest New Drugs. 2021;39(2):330–6. 32974853 10.1007/s10637-020-01015-z

[15] Meng Q, Han J, Wang P, Jia C, Guan M, Zhang B, Zhao W. BMS-794833 reduces anlotinib resistance in osteosarcoma by targeting the VEGFR/Ras/CDK2 pathway. J Bone Oncol. 2024;45:100594. 38532893 10.1016/j.jbo.2024.100594 PMC10963651

[16] El Motassime A, Vitiello R, Comodo RM, Capece G, Bocchino G, Bocchi MB, et al. Osteosarcoma: a comprehensive morphological and molecular review with prognostic implications. Biology (Basel). 2025;14(10):1407. 10.3390/biology14101407. PMID: 41154810; PMCID: PMC12561861. 10.3390/biology14101407 PMC12561861 41154810

[17] Layfield LJ, Emerson L, Crim JR, Randall L. Squamous differentiation and cytokeratin expression in an osteosarcoma: a case report and review of the literature. Clin Med Pathol. 2008;1:55–9. 21876652 10.4137/cpath.s582 PMC3160007

[18] Okada K, Hasegawa T, Yokoyama R, Beppu Y, Itoi E. Osteosarcoma with cytokeratin expression: a clinicopathological study of six cases with an emphasis on differential diagnosis from metastatic cancer. J Clin Pathol. 2003;56(10):742–6. 14514776 10.1136/jcp.56.10.742 PMC1770076

[19] Wang T, Wu X, Cui Y, Chu C, Ren G, Li W. Role of apparent diffusion coefficients with diffusion-weighted magnetic resonance imaging in differentiating between benign and malignant bone tumors. World J Surg Oncol. 2014;12:365. 25432796 10.1186/1477-7819-12-365 PMC4265400

[20] Del Grande F, Subhawong T, Weber K, Aro M, Mugera C, Fayad LM. Detection of soft-tissue sarcoma recurrence: added value of functional MR imaging techniques at 3.0 T. Radiology. 2014;271(2):499–511. 24495264 10.1148/radiol.13130844

[21] Uhl M, Saueressig U, van Buiren M, Kontny U, Niemeyer C, Köhler G, Ilyasov K, Langer M. Osteosarcoma: preliminary results of in vivo assessment of tumor necrosis after chemotherapy with diffusion- and perfusion-weighted magnetic resonance imaging. Invest Radiol. 2006;41(8):618–23. 16829744 10.1097/01.rli.0000225398.17315.68

[22] Merkle EM, Lewin JS, Aschoff AJ, Stepnick DW, Duerk JL, Lanzieri CF, Strauss M. Percutaneous magnetic resonance image-guided biopsy and aspiration in the head and neck. Laryngoscope. 2000;110(3 Pt 1):382–5. 10718423 10.1097/00005537-200003000-00009

[23] Zhu JH, Yang R, Guo YX, Wang J, Liu XJ, Guo CB. Navigation-guided core needle biopsy for skull base and parapharyngeal lesions: a five-year experience. Int J Oral Maxillofac Surg. 2021;50(1):7–13. 32536458 10.1016/j.ijom.2020.05.007

[24] Zhu JH, Liu XJ. Robotic assistance for skull base biopsy: a feasibility study in phantom and cadaver. Front Oncol. 2025;15:1669974. 41001009 10.3389/fonc.2025.1669974 PMC12457387

[25] National Comprehensive Cancer Network. NCCN Clinical Practice Guidelines in Oncology: Bone Cancer (Version 1.2021). Plymouth Meeting (PA): National Comprehensive Cancer Network; 2020. Available from: https://www.nccn.org. Cited 6 Feb 2026. 10.6004/jnccn.2026.0006 41671432

[26] Launchbury AP, Habboubi N. Epirubicin and doxorubicin: a comparison of their characteristics, therapeutic activity and toxicity. Cancer Treat Rev. 1993;19(3):197–228. 8334677 10.1016/0305-7372(93)90036-q

[27] Azizi T, Motamedi MH, Jafari SM. Gnathic osteosarcomas: a 10-year multi-center demographic study. Indian J Cancer. 2009;46(3):231–3. 19574676 10.4103/0019-509X.52958

[28] ElKordy MA, ElBaradie TS, ElSebai HI, KhairAlla SM, Amin AAE. Osteosarcoma of the jaw: Challenges in the diagnosis and treatment. J Egypt Natl Canc Inst. 2018;30(1):7–11. 29490886 10.1016/j.jnci.2018.02.001

[29] Danese MD, Groundland JS. Effect of chemotherapy and surgery timing on mortality in upper and lower extremity osteosarcoma. J Natl Cancer Inst. 2025;117(4):611–8. 39302698 10.1093/jnci/djae229

[30] Biermann JS, Chow W, Reed DR, Lucas D, Adkins DR, Agulnik M, Benjamin RS, Brigman B, Budd GT, Curry WT, et al. NCCN Guidelines Insights: Bone Cancer, Version 2.2017. J Natl Compr Canc Netw. 2017;15(2):155–67. 28188186 10.6004/jnccn.2017.0017

[31] Li H, Li Y, Song L, Ai Q, Zhang S. Retrospective review of safety and efficacy of anlotinib in advanced osteosarcoma with metastases after failure of standard multimodal therapy. Asia Pac J Clin Oncol. 2023;19(5):e314–9. 36658675 10.1111/ajco.13916

[32] Zhao D, Xie B, Yang Y, Yan P, Liang SN, Lin Q. Progress in immunotherapy for small cell lung cancer. World J Clin Oncol. 2020;11(6):370–7. 32874950 10.5306/wjco.v11.i6.370 PMC7450814

[33] Zhang RS, Liu J, Deng YT, Wu X, Jiang Y. The real-world clinical outcomes and treatment patterns of patients with unresectable locally advanced or metastatic soft tissue sarcoma treated with anlotinib in the post-ALTER0203 trial era. Cancer Med. 2022;11(11):2271–83. 35191609 10.1002/cam4.4613 PMC9160813

[34] Li Z, Fang P, Shen S, Zhang L, Xie R, Li C. Efficacy and safety of anlotinib for the treatment of advanced bone and soft tissue sarcomas: a systematic review and meta-analysis. Front Oncol. 2025;15:1703261. 41395622 10.3389/fonc.2025.1703261 PMC12698413

[35] Xu B, Pan Q, Pan H, Li H, Li X, Chen J, Pang D, Zhang B, Weng D, Peng R, et al. Anlotinib as a maintenance treatment for advanced soft tissue sarcoma after first-line chemotherapy (ALTER-S006): a multicentre, open-label, single-arm, phase 2 trial. EClinicalMedicine. 2023;64:102240. 37767191 10.1016/j.eclinm.2023.102240 PMC10520347

[36] Tang L, Niu X, Wang Z, Cai Q, Tu C, Fan Z, Yao Y. Anlotinib for Recurrent or Metastatic Primary Malignant Bone Tumor: A Multicenter, Single-Arm Trial. Front Oncol. 2022;12:811687. 35692789 10.3389/fonc.2022.811687 PMC9177947

